# Creation of a Peptide Antagonist of the GFRAL–RET Receptor Complex for the Treatment of GDF15-Induced Malaise

**DOI:** 10.1021/acs.jmedchem.3c00667

**Published:** 2023-07-28

**Authors:** Tito Borner, Ian C. Tinsley, Brandon T. Milliken, Sarah A. Doebley, Nicholas R. Najjar, Deborah J. Kerwood, Bart C. De Jonghe, Matthew R. Hayes, Robert P. Doyle

**Affiliations:** † Department of Chemistry, Syracuse University, 111 College Place, Syracuse, New York 13244, United States; ‡ Department of Biobehavioral Health Sciences, School of Nursing, University of Pennsylvania, Philadelphia, Pennsylvania 19104, United States; § Department of Psychiatry, Perelman School of Medicine, University of Pennsylvania, Philadelphia, Pennsylvania 19104, United States; ∥ Departments of Medicine and Pharmacology, State University of New York, Upstate Medical University, Syracuse, New York 13245, United States

## Abstract

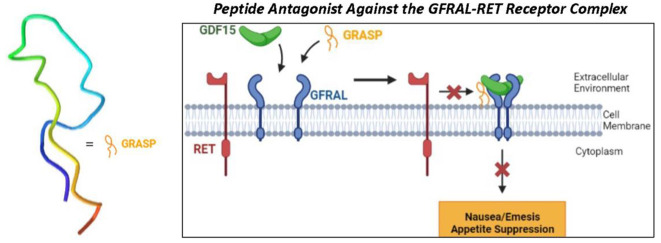

Growth differentiation factor 15 (GDF15) is a contributor to nausea, emesis, and anorexia following chemotherapy via binding to the GFRAL-RET receptor complex expressed in hindbrain neurons. Therefore, GDF15-mediated GFRAL-RET signaling is a promising target for improving treatment outcomes for chemotherapy patients. We developed peptide-based antagonists of GFRAL that block GDF15-mediated RET recruitment. Our initial library screen led to five novel peptides. Surface plasmon resonance and flow cytometric analyses of the most efficacious of this group, termed GRASP, revealed its capacity to bind to GFRAL. *In vivo* studies in rats revealed that GRASP could attenuate GDF15-induced nausea and anorexia resulting from cisplatin. Combined with Ondansetron, GRASP led to an even greater attenuation of the anorectic effects of cisplatin compared to either agent alone. Our results highlight the beneficial effects of GRASP as an agent to combat chemotherapy-induced malaise. GRASP may also be effective in other conditions associated with elevated levels of GDF15.

## Introduction

Growth differentiation factor 15 (GDF15) is a stress-response cytokine expressed by a variety of tissues that is secreted into circulation in response to a wide array of stimuli and chronic diseases, including cancer and chemotherapy.^1−4^ GDF15 signaling gained significant attention after it was confirmed to be an agonist at the GDNF family receptor α-like (GFRAL)-RET complex.^5−7^ Previously published reports highlighted the promise of GDF15-mediated signaling via GFRAL as a potential target for the pharmacologic treatment of obesity because of its potent capacity to suppress food intake.^2,8,9^ Expression of GFRAL-RET in the central nervous system (CNS) is limited to neurons in the area postrema (AP) and nucleus tractus solitarius (NTS)^5−7^ of the brainstem, where it provides critical contributions to energy balance and the induction of nausea and emesis.^10,11^ Importantly, these two adjacent structures do not possess a functional blood–brain barrier, allowing circulating GDF15 and other agents (including systemically delivered substances) to directly reach the neurons located in the AP/NTS.^12,13^ Results from several recent reports revealed that the anorectic response to GDF15 signaling was largely secondary to malaise,^14−16^ and as such the GDF15-GFRAL signaling pathway may be a potential target for the development of new antiemetic pharmacological agents. Most recently, Hsu et al.^17^ reported that GFRAL knockout mice were insensitive to the long-term anorexia and cachexia/weight loss that typically results from the administration of cisplatin. Similarly, antibody-mediated neutralization of GDF15 attenuated cisplatin-induced emesis in nonhuman primates.^18^ Taken together, there is now compelling evidence to suggest that GDF15 is a critical contributor to chemotherapy-induced nausea and vomiting (CINV) and anorexia.^19−21^ These adverse effects occur in a high percentage of cancer patients who are undergoing chemotherapy and persist despite the administration of existing antiemetic medications.^10,19,22−24^ Drugs that are prescribed ubiquitously to mitigate CINV such as serotonin receptor 3 (5-HT_3_R) antagonists are largely ineffective in preventing GDF15-induced malaise and disordered energy balance.^15^ Therefore, we hypothesize that inhibition of GDF15-mediated GFRAL-RET signaling in the hindbrain holds considerable promise as a therapeutic target to achieve the control of nausea, emesis, and anorexia in patients undergoing chemotherapy.

As a first step in this direction, we developed a peptide antagonist (GRASP) that blocks signaling at GFRAL and thus attenuates GDF15- and chemotherapy-induced malaise. GRASP was one of the five peptides that were initially identified from *in silico* modeling and docking simulation experiments. All five peptides were generated by solid-phase synthesis; each included an azido-modified lysine residue to facilitate site-selective conjugation using click chemistry. Surface plasmon resonance (SPR) binding experiments were performed to assess the binding affinity (*K*_D_) of these peptides to GFRAL. The 29-amino acid peptide, GRASP, exhibited the strongest binding affinity (179 μM) and was thus selected for further evaluation. We generated conjugated fluorescent sulfo-Cy5 and Alexa Fluor 546 GRASP conjugates for use in *in vitro* flow cytometry and *in vivo* tracking experiments, respectively. The solution state structure of GRASP determined by nuclear magnetic resonance (NMR) and restrained molecular dynamics revealed secondary hairpin-like motifs that were similar to the loops observed in GDF15 (BMRB accession number 51672). Initial proof of concept *in vivo* studies revealed high levels of GRASP colocalization with GFRAL expressed by neurons in the rat AP and NTS. We then examined the impact of central and systemic GRASP administration on both cisplatin- and GDF15-induced malaise in rats. Finally, we combined GRASP with the 5-HT_3_ antagonist, Ondansetron, to determine whether combination treatment resulted in greater improvements in cisplatin-induced malaise than could be achieved with either agent administered alone.

## Results and Discussion

### Design and Synthesis of GRASP

Using a structure-based rational design strategy that focused on previously reported^17^ interactions of GDF15 with the GFRAL–RET receptor complex, an initial small library of ∼10 peptides was designed. Regions of GDF15 identified as critical for GFRAL receptor specificity were incorporated in the peptides in this initial library, together with regions in selected members of the TGF-β superfamily that had been identified as important for the GDNF family receptor binding. Based on our analysis of GDNF family receptors, notably GDNFα1 and GDNFα3, we identified regions of sequence homology across the receptor family that were essential for binding to cognate receptors. We incorporated these homologous regions into the design and synthesis of the peptides included in our library. Initial design strategies focused on interactions with GFRAL residues 201–203 (serine–lysine–glutamate, or SKE), which are unique to GFRAL and replaced the “RRR” motif identified as critical for selectivity in other TGF-β receptors.^17^ Finally, rather than designing a peptide to compete directly with GDF15, we aimed to prevent RET recruitment by replacing critical GDF15 residues (e.g., W32)^5,17^ with bulky hydrophilic residues. We also incorporated sequences capable of binding metal ions (particularly zinc) to block this interaction. Library peptides were synthesized via a solid-phase system by using a microwave-assisted CEM Liberty Blue peptide synthesizer. Azido-modified lysine residues were included in each sequence to facilitate future conjugations needed for mechanistic studies (Supporting Information, (SI), Table S1).

### Evaluation of Peptide Binding to GFRAL (S19–E351) with Surface Plasmon Resonance (SPR)

**Figure 1:**
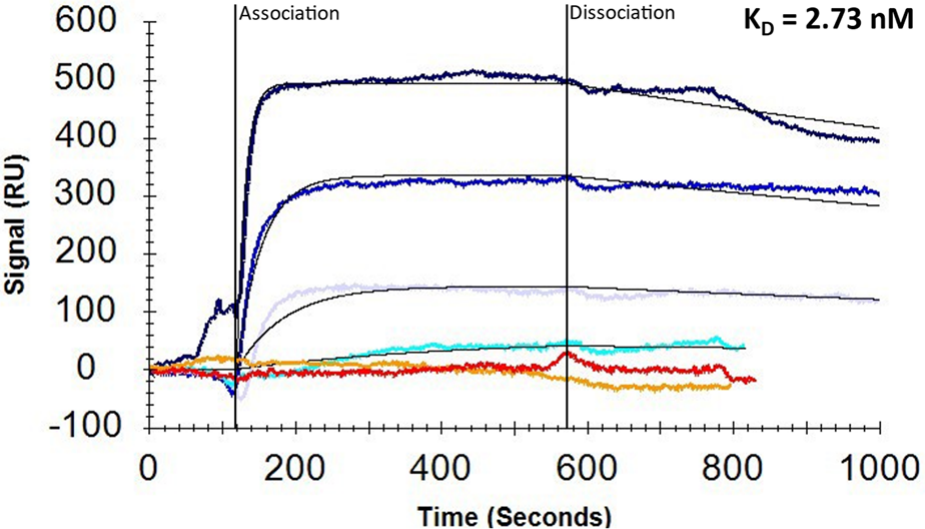
Surface plasmon resonance (SPR) with biotin-conjugated GRASP shown in dose–response sensorgrams with overlaid TraceDrawer kinetic evaluation curves generated using a 1:1 local *B*_max_ fit. Results from increasing concentrations of GFRAL (20, 100, 200, and 600 nM) are displayed in different shades of blue with GDF15 (1 μM) in red and RET (0.29 μM) in orange.

Peptide binding to a segment of recombinant human GFRAL containing the complete known binding domain of GDF15 and the remaining extracellular domain (S19–E351) of the receptor as defined by UniProt (Q6UXV0) was evaluated by SPR (Figure 1). Initial screening aimed to confirm GFRAL immobilization and system functionality by evaluating the dose–response profile to a commercial formulation of HEK293 cell-derived recombinant human GDF15. Our initial evaluation of GDF15 generated a binding affinity (*K*_D_) of 6.02 nM, which is consistent with values reported in the literature^6^ and thus validated our in-house approach (SI, Figure S8). For example, Yang et al.^6^ reported a *K*_D_ of ∼1 nM for the interaction between untagged GDF15 and purified human GFRAL extracellular domain (ECD) and binding of GDF15 to HEK293 transfectants expressing human GFRAL-EDC (Ser19–Glu351) and GDF15 via SPR and flow cytometry, respectively.

The binding of each peptide to the ECD of GFRAL was initially confirmed from the results of carboxyl-sensor SPR (SI, Figure S9) with bound GFRAL–ECD. A broad range of binding affinities (*K*_D_ values ranging from 0.179 to 64.8 mM) was observed. While GRASP was the only peptide to demonstrate submillimolar binding affinity (179 μM) to GFRAL–ECD, this interaction was weaker than that observed for the GDF15 control (6 nM). We hypothesized this may be due to obstruction of the binding site of GFRAL–ECD in its immobilized state. Thus, we opted for a different SPR approach in which we conjugated GRASP to biotin and immobilized the peptide to the sensor surface, this time via biotin–streptavidin affinity. Increasing concentrations of GFRAL–ECD were then evaluated as the mobile phase against the immobilized GRASP. This approach yielded a substantial increase in the observed binding affinity (74.1 nM; Figure 1). Additionally, neither RET nor GDF15 controls showed binding of such to GRASP. We then proceeded to explore GRASP binding *in vitro* by flow cytometry using a fluorescently labeled ligand and stably transfected GFRAL-expressing HEK293 target cells.

### Synthesis of Fluorescently Labeled GRASP

**Figure 2:**
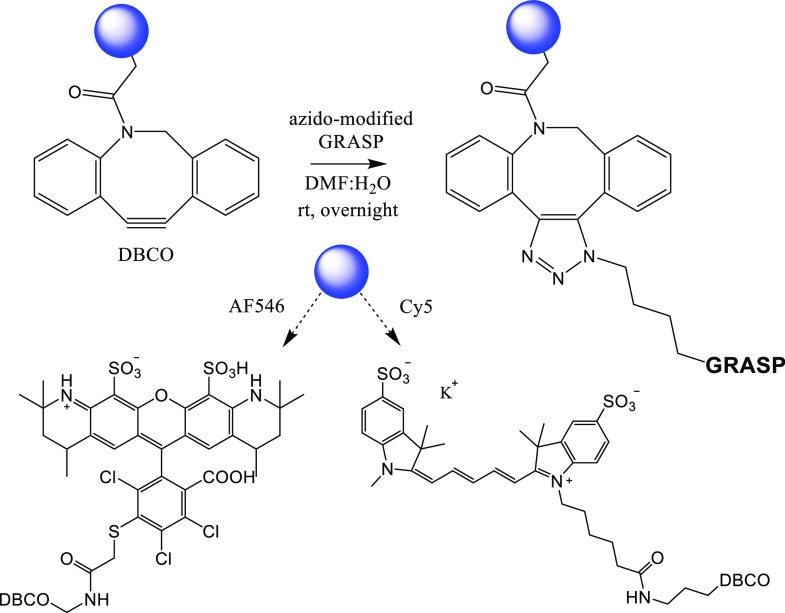
Synthesis of fluorescently conjugated GRASP555 and GRASPCy5. The 29 amino-acid azido-modified (K2-position) GRASP peptide was linked to the fluorescent-DBCO moiety via standard copper-free alkyne–azide cycloaddition, which resulted in stoichiometric yields of a 1:1 fluorophore:GRASP conjugate at the K2 peptide residue.

Two fluorescently labeled GRASP conjugates (GRASPCy5 and GRASP555) were synthesized by strain-promoted alkyne–azide cycloaddition (SPAAC) (Figure 2). Initially, we utilized GRASP555 in experiments (as noted specifically throughout) but switched in later experiments to GRASPCy5 due to better fluorescence stability and ease of purification. Fluorescent conjugates are widely used as probes for the study of subcellular protein localization and gene expression as well as in biosensing applications. Fluorophores spontaneously emit light at specific wavelengths and require no exogenous substrates. SPAAC is a versatile tool for site-selective conjugation to a broad range of substrates used to study peptide-based therapeutics. We utilized fluorescently labeled GRASP for *in vitro* surface recognition studies targeting GFRAL expressed in HEK293 cells as well as *in vivo* colocalization with GFRAL-expressing neurons in the hindbrain.

### Evaluation of Interactions of GRASPCy5 with HEK293 Cells Stably Expressing GFRAL *in Vitro* by Flow Cytometry

**Figure 3:**
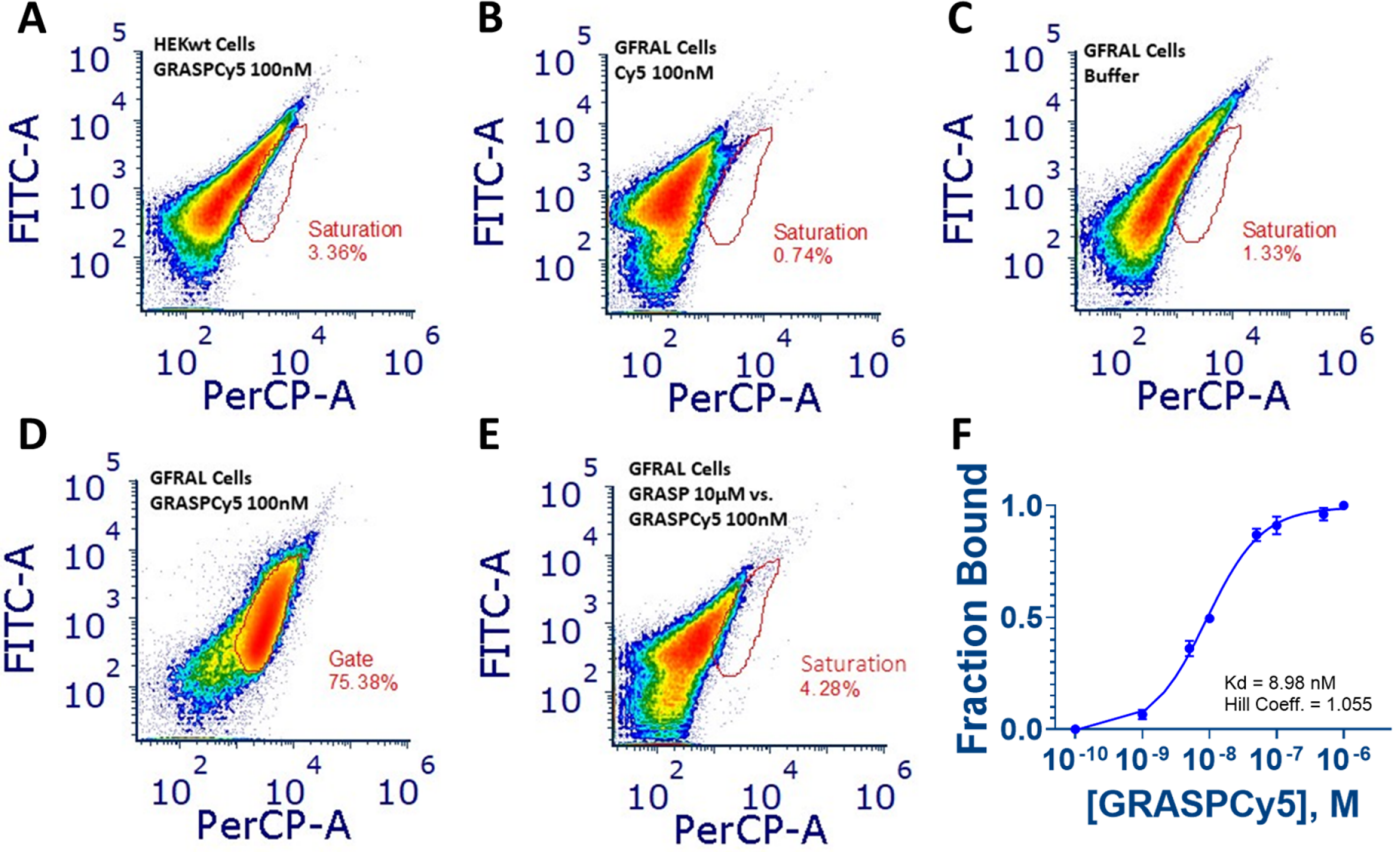
Flow cytometric analysis of GFRAL–HEK293 cells probed with GRASPCy5. (A) Control (untransfected) HEK293 cells with 100 nM GRASPCy5, (B) GFRAL–HEK293 cells treated with 100 nM Cy5 (unconjugated), (C) GFRAL–HEK293 cells probed with buffer control, (D) GFRAL–HEK293 probed with 100 nM GRASPCy5, (E) GFRAL–HEK293 cells probed with 100 nM GRASPCy5 in the presence of 10 10 μM unlabeled GRASP, and (F) fraction bound vs GRASPCy5 concentration (M) at GRASPCy5 concentrations of 0.1, 1, 5, 10, 50, 100, 500, and 1000 nM; *K*_D_ = 8.98 nM with a Hill coefficient of 1.055.

Increasing concentrations of GRASPCy5 were added to cultures of stably transfected HEK293 cells that overexpress full-length GFRAL. GRASPCy5 binding was evaluated by flow cytometry and compared with results from untransfected HEK293 cells. Only GFRAL-expressing HEK293 cells exhibited a significant shift upon the addition of GRASPCy5 (Figure 3); these findings revealed that GRASPCy5 interacted with GFRAL-expressing, but not untransfected HEK293 cells. The dose–response curve elicited by GRASPCy5 generated a *K*_D_ of 8.98 nM (Figure 3F), which is comparable to the reported *K*_D_ of 1 nM for the native ligand and is in line with the SPR data generated herein (Figure 1). Of note, this is substantially higher than the *K*_D_ calculated from the results of the SPR experiments on the carboxyl chips, which is consistent with the notion that GRASP does not bind to the same binding site as GDF15 in the GFRAL–ECD, one likely inhibited by GFRAL immobilization on the carboxyl sensor.

### Solution Structure of GRASP Determined by Nuclear Magnetic Resonance and Restrained Molecular Dynamics

**Figure 4:**
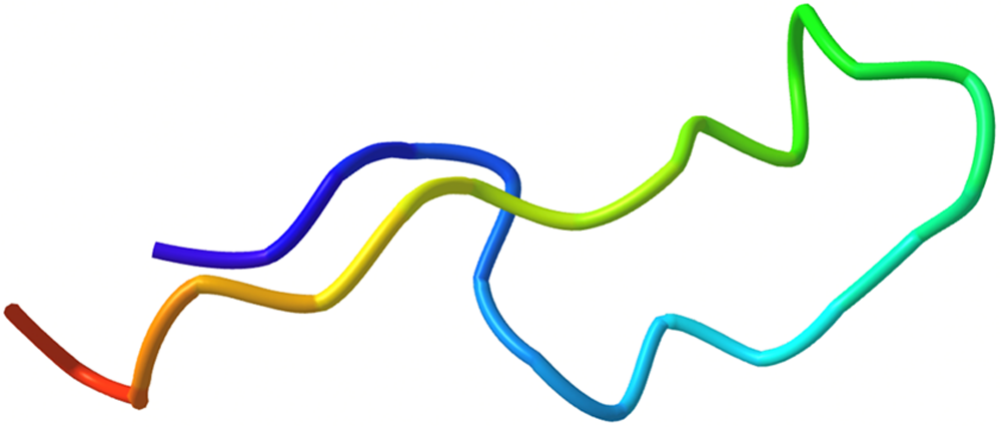
Solution-state structure of GRASP [T(K-azido)EELIHAHADPMVLIQKTDTGVSLQTYD; 300 μM, pH 6.8 in 50 mM PBS buffer spiked with 10% D_2_O, 800 MHz NMR with a cryoprobe at 25 °C] as a snapshot based on MD simulations, which reveals a secondary hairpin-like structure with a GDF15-like loop. This structure was the result of a 15 ns implicit solvent AMBER18 run^25,26^ in which NMR nuclear Overhauser enhancement (NOE)-based distance restraints and TALOS+-based PHI and PSI angle ranges were incorporated.^27^ Structure is deposited in the Biological Magnetic Resonance Databank (BMRB) as accession number 51672.

**Figure 5:**
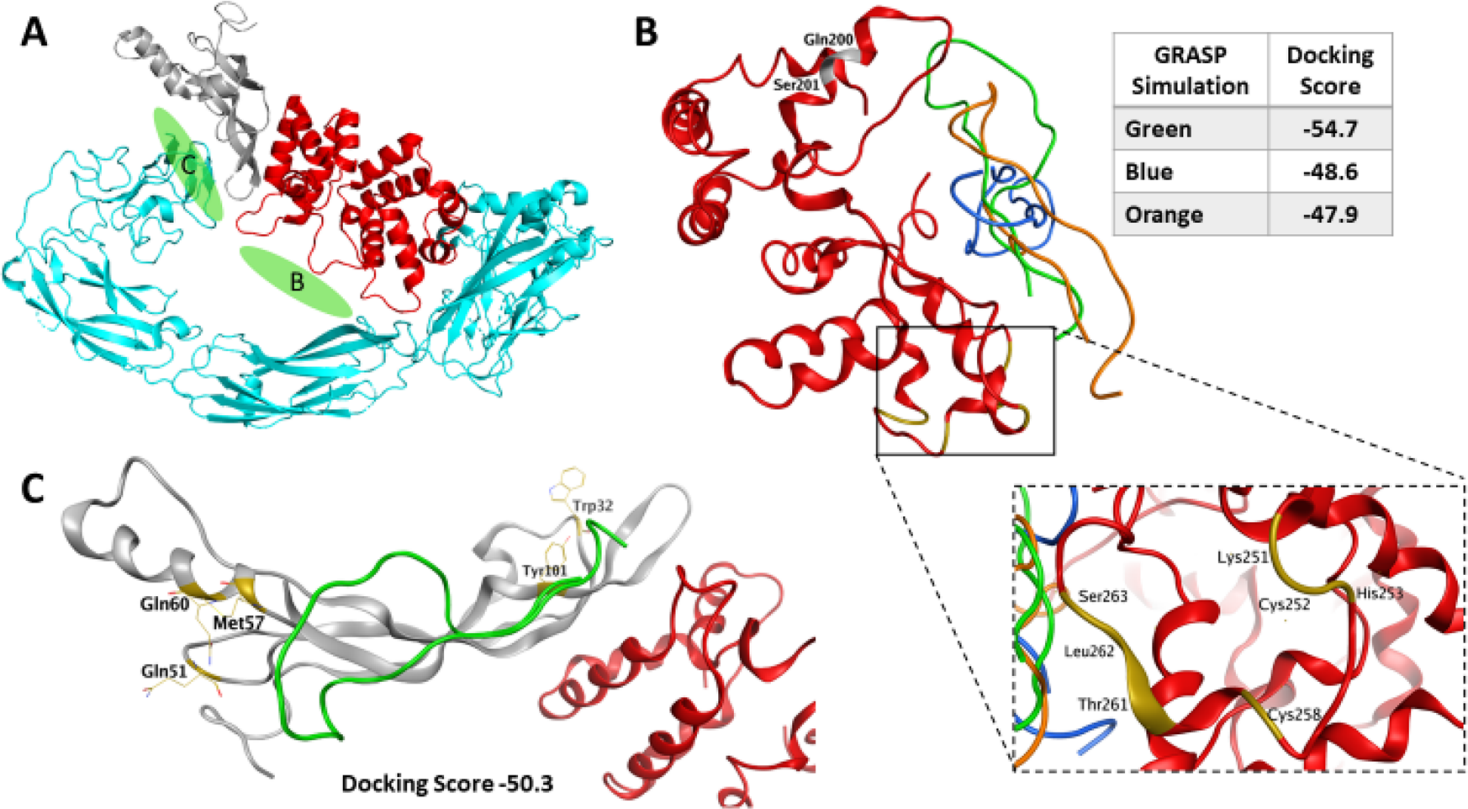
GRASP docking to GFRAL and GFRAL–GDF15. (A) The CryoEM structure of GFRAL–GDF15-RET complex (PDB 6Q2J) was used to generate an *in silico* prediction of GRASP docking (green; described herein as NMR solution structure (BMRB 51672); see also Figure 6); shown are GFRAL (red), GDF15 (gray), and RET (cyan).^28^ (B) GFRAL extracellular domain (ECD) (Trp129–Asn318; red, PDB 5VZ4) with the GRASP NMR structure in bound configuration displaying the top three MOE calculations and corresponding docking scores. Gray-colored GFRAL residues are those directly involved in GDF15 binding; gold-colored residues are those directly involved in RET recruitment to the GFRAL–RET interface. (C) GDF15 (gray) bound to GFRAL (red) (PDB 5VZ4) displaying the preferential docking domain of GRASP to the GFRAL–GDF15 complex; gold-colored residues are those directly involved in RET recruitment to the GDF15–RET interface. Docking model PDB is supplied as Supporting Information.

NMR-based restrained molecular dynamics was used to determine the solution structure of GRASP, which revealed a loop region with a hairpin-like fold (Figure 4). We took a high-ambiguity driven protein–protein docking modeling approach using Molecular Operating Environment (MOE) software to dock the constrained NMR solution structure of GRASP (BMRB accession number: 51672) (SI, Figure S11–13) on the GFRAL–ECD (PDB 5VZ4) residues Trp129–Asn318, both in the presence and absence of bound GDF15 (Figure 5).

**Figure 6:**
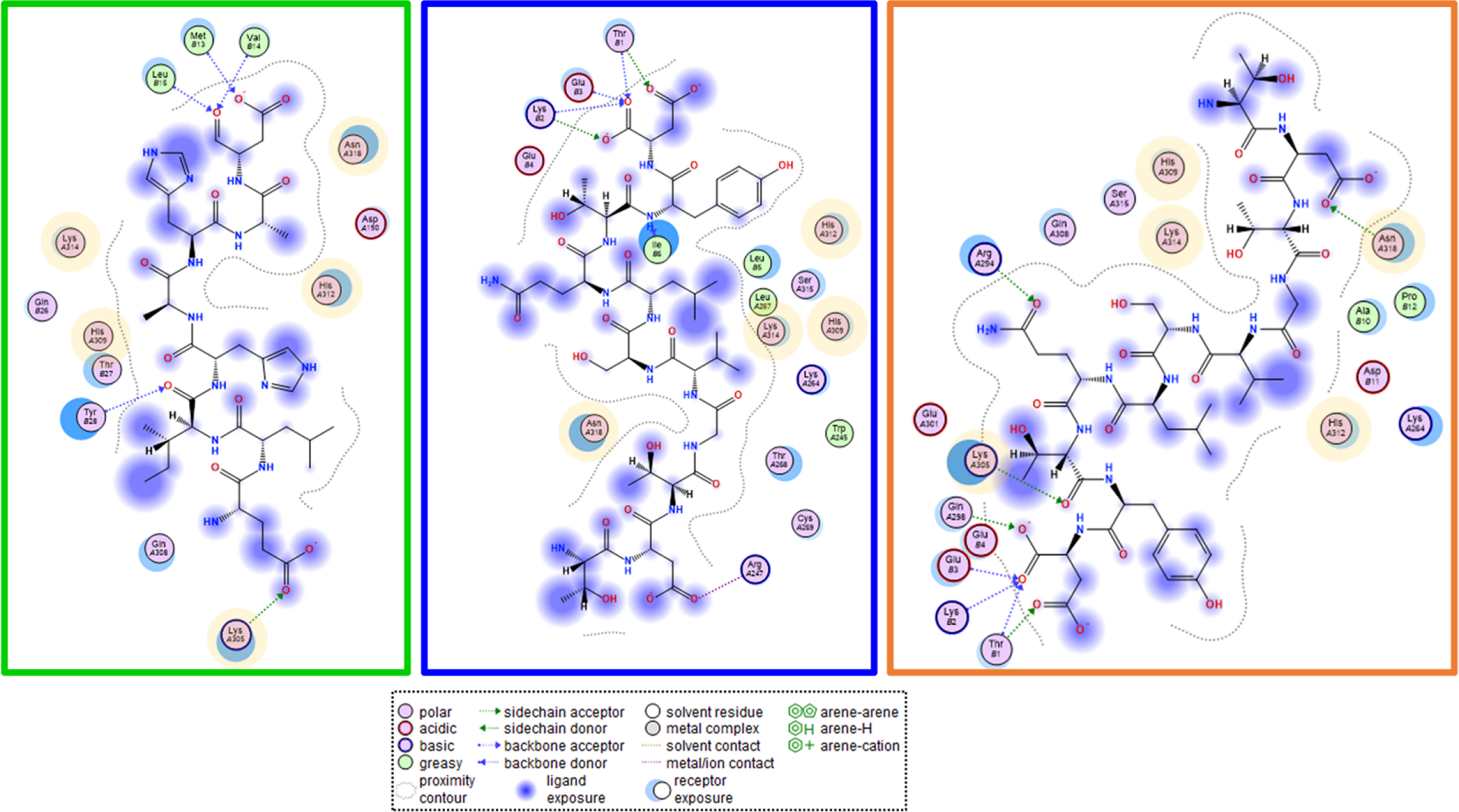
Representative GRASP binding pockets for the top three calculations from GFRAL docking studies are shown in Figure 5B. Conserved GFRAL residues involved in GRASP recognition are highlighted. It should be noted that the HAHA motif of GRASP is depicted in the green box, displaying the best docking score.

We noted *in silico* that GRASP had a preferential docking pocket proximal to the binding site for GDF15 (Figure 5B), consistent with the SPR data observations; the top three calculations included docking in the direction of recruitment of RET to the GFRAL–GDF15 complex (Figure 5A). Furthermore, GRASP displayed an additional docking domain with relatively similar docking scores at the GDF15 interface with RET recruitment that appears to block access to multiple residues that have been directly implicated in binding of RET to GDF15 (Figure 5C). Further evaluation of the docking of GRASP to the ECD of GFRAL revealed that several residues involved in GRASP recognition were conserved (Figure 6). Each GRASP terminus displays interactions (shown in green) with residues near the N-terminal domain; these include a HAHA motif (shown in blue and orange) docking via the C-terminal domain. MOE calculations suggest that GRASP is a noncompetitive antagonist that can inhibit RET recruitment via multiple binding interactions with GFRAL that serve as direct impediments at the RET–GDF15 interface of the GFRAL–GDF15 complex.

### *Ex Vivo* Colocalization of GRASP555 with *GFRAL*-Positive Neurons in the Rat AP/NTS

**Figure 7:**
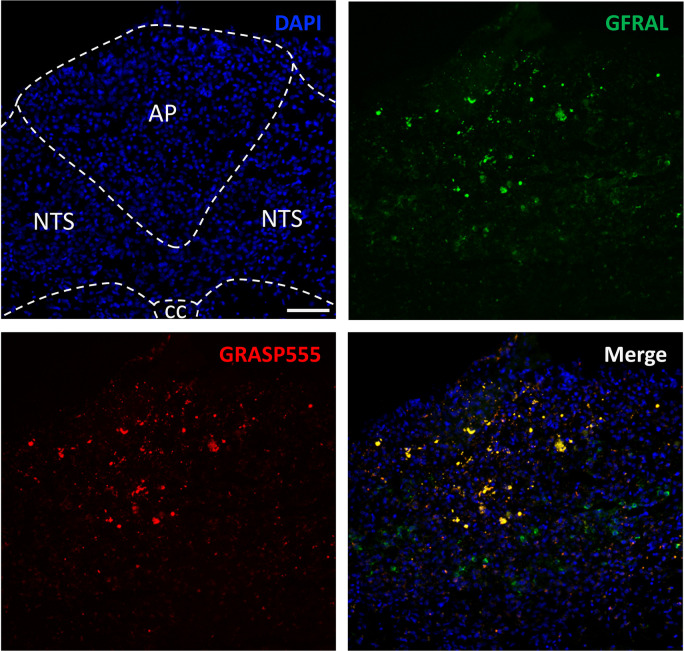
Representative images of the AP and the medial NTS documenting high levels of colocalization of fluorescently tagged GRASP (GRASP555) with unlabeled GFRAL. GRASP555 (300 pmol in 1 μL) was injected into the lateral ventricle of wild-type rats 2 h before sacrifice. Brain tissues were then removed and processed for immunohistochemistry. AP, area postrema; NTS, nucleus tractus solitarius; CC, central canal. Scale bar: 100 μm.

As a first proof of concept *in vivo*, we determined whether GRASP555 would colocalize with GFRAL-positive neurons in the AP/NTS. GRASP555 was delivered directly to the CNS of experimental rats via lateral ventricle infusion to determine whether this molecule would retain its ability to bind to the GFRAL receptor *in vivo* (Figure 7). Our proof-of-concept microscopy experiment revealed prominent colocalization of fluorescently tagged GRASP555 with GFRAL (labeled with a specific primary and Alexa Fluor 488-conjugated secondary antibody) in the AP and NTS of the rat. Little to no GRASP555 fluorescence was detected within regions of the hindbrain that do not express GFRAL. While dose–response and multiple time-point studies employing systemic administration of GRASP555 are needed to fully characterize its biodistribution, these current results collectively corroborate the specificity of GRASP for GFRAL and highlight the successful binding of this peptide to GFRAL–RET both *in vitro* and *in vivo*.

### Systemic and CNS Administration of GRASP Attenuates GDF15-Induced Malaise in Rodents

**Figure 8:**
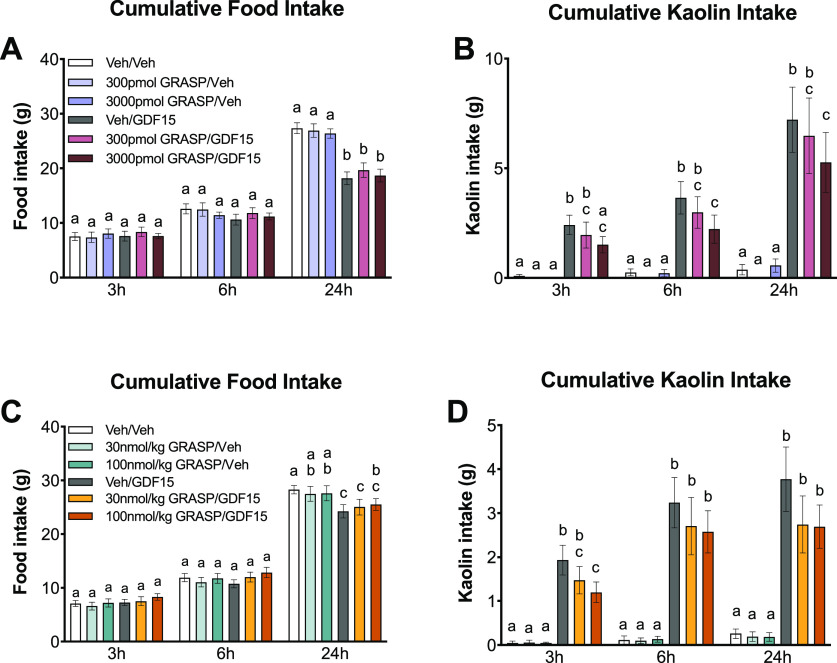
(A) In rats, anorexia induced by exogenous GDF15 (20 μg/kg, IP) was not affected by intraventricular administration (4th ICV) of GRASP (*n* = 10 per group). (B) GRASP 4th ICV administration elicited dose-dependent attenuation of GDF15-induced kaolin consumption, a well-validated proxy for emesis and nausea in rats (GDF15 at 20 μg/kg, *n* = 10 per group). (C) Systemic administration of GRASP failed to attenuate GDF15-induced anorexia in rats (GDF15 at 20 μg/kg IP, *n* = 12 per group). (D) The highest dose of GRASP tested (100 nmol/kg, i.e., 328 μg/kg) significantly attenuated kaolin intake resulting from systemic delivery of GDF15 (20 μg/kg IP, *n* = 12/group). Data were analyzed with repeated measure 2 × 3 ANOVAs followed by Tukey *post hoc* tests. All data are expressed as means ± standard error of the mean (SEM). Means with different letters are significantly different from each other (*P* < 0.05).

In initial experiments, we examined whether central administration of GRASP (i.e., directly into the fourth ventricle or fourth ICV) would counteract the effects of centrally delivered GDF15 in rats. Because the AP/NTS is located on the dorsal surface of the hindbrain at the caudal end of the fourth ventricle, this route of administration facilitates targeted and selective drug delivery near the sites of GFRAL expression. To assess the impact of these interventions, we measured pica behavior, i.e., the ingestion of non-nutritive substances such as kaolin,^29^ as this represents an accurate and reliable proxy for malaise in rats and has been used to examine the effects of agents known to cause nausea in humans.^29−31^ Consistent with our previous studies, central administration of GDF15 (30 pmol) induced significant kaolin consumption and anorexia compared to vehicle injections (Figure 8A,B). While no specific effects were observed in response to central injections of GRASP alone at all doses tested, pretreatment with GRASP reduced GDF-induced kaolin intake in a dose-dependent fashion (Figure 8A,B).

To increase the translational impact of this study and more closely mimic a potential clinical scenario, we conducted a dose–response study in which both GRASP and GDF15 were administered systemically to rats via the intraperitoneal (IP) route. The selected dose of GDF15 (20 μg/kg) mimics endogenous levels of GDF15 observed following chemotherapy treatment^14,15^ and reliably induced kaolin consumption and modest, albeit statistically significant anorexia (Figure 8C,D). By contrast, systemic (IP) administration of GRASP alone had no impact on food and kaolin intake. Although systemic GRASP administration failed to attenuate GDF15-induced anorexia in rats (Figure 8C), the highest dose of GRASP tested (100 nmol/kg) significantly attenuated kaolin intake induced by systemically delivered GDF15 (Figure 8D).

### Systemic Administration of GRASP Attenuates Chemotherapy-Induced Malaise and Coadministration with Ondansetron Attenuates Chemotherapy-Induced Anorexia in Rats

**Figure 9:**
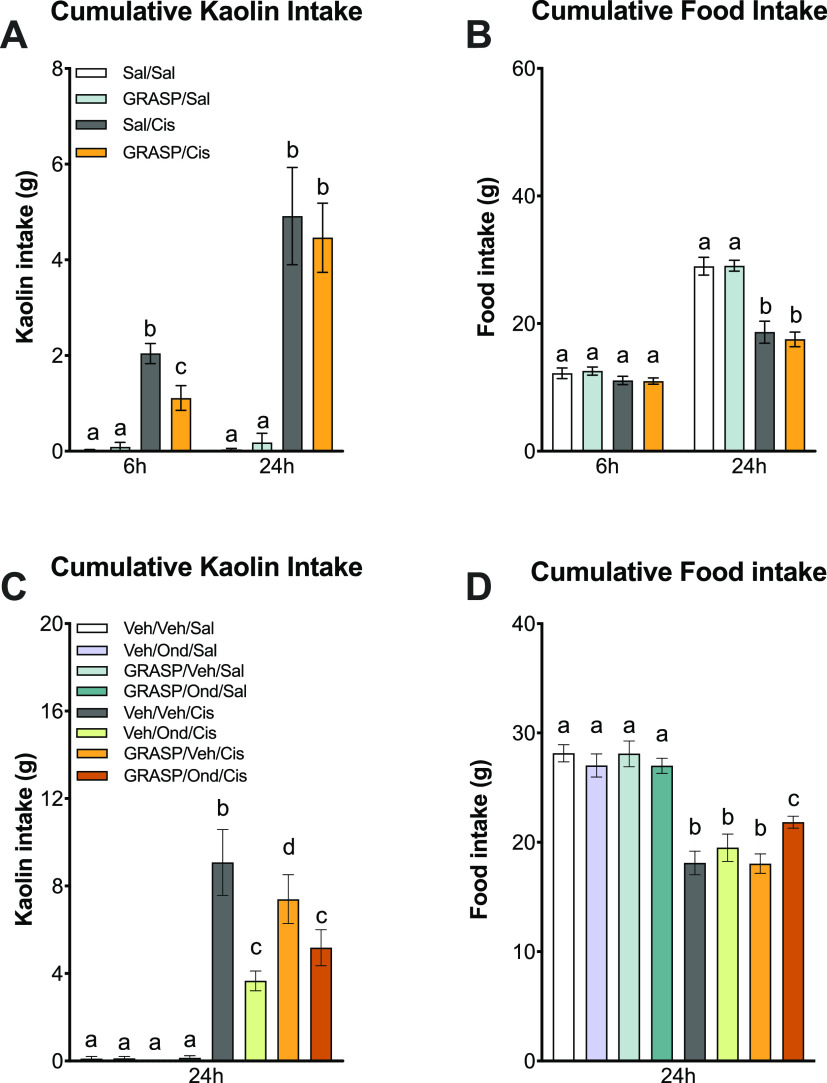
(A) Kaolin intake induced by a single dose of the highly emetogenic chemotherapeutic agent cisplatin (6 mg/kg IP) was significantly attenuated at 6 h by GRASP treatment (100 nmol/kg IP, *n* = 7–10 per group). (B) Cisplatin-induced anorexia was not attenuated by GRASP (100 nmol/kg of IP, *n* = 7–10 per group). (C) Administration of Ondansetron (Ond; 2 mg/kg IP) or GRASP (100 nmol/kg IP twice per day [BID]) alone significantly reduced kaolin intake included by cisplatin, although no additive effect was observed in response to simultaneous administration of both drugs (*n* = 10–16 per group). (D) Neither Ond (2 mg/kg of IP) nor GRASP (100 nmol/kg of IP [BID]) alone had any impact on cisplatin-induced anorexia. By contrast, simultaneous treatment with both drugs led to a significant attenuation of cisplatin-induced anorexia (*n* = 10–16/group). Data shown in parts A and B were analyzed with 2 × 2 ANOVAs followed by Tukey *post hoc* tests. Data in parts C and D were analyzed with one-way ANOVAs followed by Tukey *post hoc* tests. All data are expressed as means ± SEMs. Bars were labeled with different letters (a, b, and c) to indicate significant differences from one another (*p* < 0.05).

In this set of experiments, rats were treated with the highly emetogenic chemotherapeutic agent, cisplatin. Using this gold-standard preclinical model of CINV, we first determined the effects of a single systemic dose of GRASP administered shortly before cisplatin. Consistent with our previous studies and with findings published in the literature,^32,33^ a single dose of cisplatin leads to significant kaolin consumption and anorexia in rats compared to controls (Figure 9A,B). While systemic administration of GRASP alone (100 nmol/kg IP) had no effect on these outcomes, pretreatment with GRASP (i.e., 15 min prior to cisplatin injection) reduced kaolin intake induced by cisplatin administration at the 6 h time point (Figure 9A,B).

In a follow-up experiment, we evaluated responses to the gold standard antiemetic Ondansetron alone or in combination with GRASP to determine whether the combination would enhance the efficacy of the former compound in preventing CINV in rats. We found that GRASP alone or in combination with Ondansetron was well-tolerated and had no impact on food or kaolin intake in healthy rats. As anticipated, cisplatin-induced both anorexia and significant kaolin intake **(**Figure 9C,D). While administration of GRASP or Ondansetron (100 nmol/kg of IP BID and 2 mg/kg of IP, respectively) significantly reduced cisplatin-induced kaolin intake, no additive effect was observed in response to dual treatment (Figure 9C). By contrast, significant attenuation of cisplatin-induced anorexia was observed in rats receiving combined GRASP and Ondansetron treatments compared to the responses observed to each treatment alone (Figure 9D). Overall, the results of these studies demonstrate the antiemetic properties of GRASP and thus its value as a potential treatment for CINV when combined with current antiemetic medications such as Ondansetron.

In general, the effects of GRASP on malaise (i.e., kaolin consumption) were stronger than those observed on food intake. Most likely, the lack of an effect of a single dose of GRASP on GDF15-induced anorexia is due to the different temporal profile of these two behavioral phenomena combined with the half-life of the GRASP peptide. Kaolin intake in rats manifests relatively quickly after GDF15 and cisplatin administrations, respectively. In contrast, the onset of GDF15- and cisplatin-induced anorexia occur several hours thereafter.^15,32,33^ It is therefore plausible that at these later time points GRASP is no longer present in sufficiently high concentrations to prevent anorexia. It is also worth mentioning that CINV involves multiple mediators/systems, including but not limited to serotonin and substance P^10^, which are not affected by GDF15 (and hence GRASP).

## Conclusions

CINV is a highly prevalent condition associated with poor quality of life and reduced survival of patients undergoing cancer treatment. While the pharmacological management of CINV has improved, chemotherapy-induced malaise remains poorly controlled and poorly understood. The findings presented here illustrate the importance of potential mediators of nausea and anorexia, such as GDF15, in our efforts to identify better strategies for CINV control. GDF15 has been identified as a critical mediator of CINV; elevated circulating GDF15 concentrations correlate with cachexia and reduced survival in patients diagnosed with cancer.^34,35^ Importantly, none of the current FDA-approved antiemetics used in the oncology field successfully counteracted GDF15-induced anorexia and malaise in rats,^32^ suggesting that GDF15 may account for the lack of complete CINV control in patients, thus stressing the importance of developing additional treatments to effectively block GDF15 effects. In this article, we describe the creation and characterization of GRASP, a peptide molecule that targets GFRAL and inhibits RET signaling by preventing its GDF15-driven interaction with GFRAL on the cell surface. Importantly, GRASP administration attenuates both GDF15- and cisplatin-induced malaise in rats.

Collectively, our results highlight the beneficial effects of GRASP treatment and its potential for future use as a novel treatment of chemotherapy-induced malaise and potentially other conditions associated with uncontrolled nausea and vomiting due to elevated GDF15 levels (e.g., hyperemesis gravidarum). In this regard, the literature suggests that women are more susceptible to CINV compared to men.^34,36^ Although exploring the potential link between GDF15 and its sex-specific effects and, subsequently, comparing the beneficial actions of GRASP in male and female rats is beyond the scope of the current manuscript, it remains nonetheless an intriguing and clinically relevant venue for further research.

The design of a noncompetitive antagonist of the GFRAL–RET receptor is a unique approach in the important and rapidly growing research area of GDF15–GFRAL–RET signaling. To the best of our knowledge, GRASP is currently the only peptide antagonist of its kind and is a promising first approach toward antagonizing the GFRAL–RET complex to treat GDF15-driven nausea/emesis and malaise. The work performed thus far also sheds light on our fundamental understanding of how GDF15 induces reductions in food intake and body weight. For example, we showed that GRASP administered intraventricularly could penetrate the AP/NTS and colocalize with GFRAL receptors. Thus, GRASP has the potential to mitigate several undesirable GDF15-induced sickness behaviors, including those resulting from GDF15 produced and released from cells in the CNS. While GRASP shows potential as a stand-alone therapeutic, it may also be effective when paired with other compounds to mitigate the adverse effects of currently approved therapeutics. Novel agents of this class with the potential to address this clinically unmet need will also advance the fields of ingestive behavior and obesity research and may help patients tolerate metabolic disease treatments via their impact on the GFRAL–RET complex.

## Experimental Section

### General Materials

Novel peptides (GRASP and GSPs) were produced either by Genscript (Piscataway, NJ) or in-house using a microwave assisted CEM Liberty Blue peptide synthesizer. Peptides were synthesized with an azido-modified lysine within the N-terminal domain to facilitate future bioconjugation. Sequences were confirmed by MS/MS and purity was determined by Shimadzu Prominence high-performance liquid chromatography (HPLC) using a 10–90% HPLC grade acetonitrile gradient for 25 min at a flow rate of 1 mL/min over an Agilent Eclipse XDB-C18 column (5 μm, 4.6 mm × 150 mm, PN 993967-902) tracked at 280 nm. Peptides were purified to >95% and identified via MS on either a Shimadzu Triple-Quad LCMS-8040 or MALDI-TOF MS (SI, Figure S1). An in-house formulation of Hank’s Balanced Salt Solution (HBSS), pH 7.4, was used in both the binding and surface recognition experiments and was generated as follows: NaCl, 140 mM, (ThermoScientific, 447300050, lot no. A0437721); KCl, 5 mM, (Sigma Life Sciences, P5404-550G, lot no. SLBW2757); CaCl_2_, 1 mM (Sigma-Aldrich, C5670-500G, lot no. SLCL2092); MgSO_4_·7H_2_O, 0.4 mM (Sigma-Aldrich, M2773–500G, lot no. SLCN3621); MgCl_2_·7H_2_O, 0.5 mM (Sigma Life Sciences, M2393-500G, lot no. SLBT5995); Na_2_HPO_4_, 0.3 mM, (Sigma-Aldrich, S5136–500G, lot no. BCCH2211); KH_2_PO_4_, 0.4 mM, (Sigma-Aldrich, P9791-1KG, lot no. SLCK2007); NaHCO_3_, 4 mM (Amresco, 0865-1KG, lot no. 2013C347); and d-glucose, 6 mM (Sigma Life Sciences, G7021-100G, lot no. SLBX8648).

### Solid-Phase Peptide Syntheses

Solid-phase peptide synthesis was performed using a microwave-assisted CEM Liberty Blue peptide synthesizer (Matthews, NC, USA) on a ProTide Rink amide resin. Fmoc-protected amino acids were coupled to the resin using Oxyma Pure (0.25 M) and *N*,*N*′-diisopropyl carbodiimide (0.125 M) (Sigma-Aldrich, 38370-25 ML, lot no. BCCF5927) as the activator and activator base, respectively. Fmoc protection groups were removed between couplings with 20% piperidine (Sigma-Aldrich, 110–89–4). Global deprotection and cleavage of the peptides from the solid-support resin were achieved using a CEM Razor (Matthews, NC, USA) via a 30 min incubation at 40 °C in a mixture of 95% trifluoroacetic acid (Sigma-Aldrich, 76–05–1), 2.5% triisopropylsilane (Sigma-Aldrich, 233781-250G, lot no. MKCL3354), and deionized (DI) water. Peptides were precipitated with diethyl ether at 4 °C, purified, and confirmed by RP-HPLC and MS, respectively.

### Synthesis of GRASP555

Dibenzocyclooctyne (DBCO) modified AlexaFluor546 (DBCO-AF546, Jena Bioscience, CLK-1286) was conjugated to K2-azido modified GRASP by dissolving in 4:1 dimethylformamide (DMF):H_2_O with stirring overnight at room temperature. The resulting 1:1 GRASP(K2)–Fluorophore conjugate (GRASP555) was RP-HPLC-purified to 99% in stoichiometric yields (SI, Figure S2). ESI-MS expected *m*/*z* = 4386, observed *m*/*z* [M + 3H]^+3^: 1463, [M + 4H^+^ + H_2_O]^+4^: 1116, [M + 4H]^+4^: 1097, and [M + 5H]^+5^: 878 (SI, Figure S3). Successfully linkage of GRASP to DBCO-AF546 resulted in a shift of the excitation-maxima shift from 554 to 560 nm and of the emission maxima from 570 to 571 nm (SI, Figure S4).

### Synthesis of GRASPCy5

GRASPCy5 was generated by conjugating GRASP to DBCO-modified sulfocyanine5 (DBCO-Cy5, Lumiprobe, 233F0) by dissolving it in 9:1 DMF: H_2_O followed by stirring overnight at room temperature, resulting in a 1:1 GRASP(K2)–fluorophore conjugate (GRASPCy5), which was RP-HPLC to 95% purity at stoichiometric yields (SI, Figure S5). MALDI-TOF-MS results included: expected *m*/*z* = 4260 Da, observed 4243 Da, −K^+^, +Na^+^ (SI, Figure S6). Successfully linkage of GRASP to DBCO-Cy5 resulted in a shift of the excitation-maxima from 646 to 649 nm and a shift of the emission maxima from 662 to 665.5 nm (SI, Figure S7).

### Synthesis of Biotinylated GRASP

PEG2-C4-alkyne-modified biotin molecules were conjugated to GRASP at the K2-azido residue only and at 1:1 stoichiometry, in the presence of 2.0 mol % CuI and a slight excess of 5.0 mol % tris(benzyltriazolylmethyl)amine (TBTA) dissolved in 4:1 DMF:H_2_O with stirring overnight at room temperature. Biotinylated-GRASP was purified by RP-HPLC with 95% purity. MALDI-TOF-MS: expected *m*/*z* for 1:1 biotinylated GRASP = 3747 Da, observed 3748 Da.

### NMR Solution Structure of GRASP (Deposited in BMRB as Accession Number 51672)

NMR solution structure experiments were performed on GRASP (300 μM; pH 6.8 in 50 mM PBS buffer spiked with 10% D_2_O) using an 800 MHz Bruker instrument at the SUNY College of Environmental Science and Forestry with a cryoprobe at 25 °C. To assign the peaks, the following experiments were performed: Nuclear Overhauser effect spectroscopy (NOESY) using excitation sculpting for water suppression. The spectral width in both dimensions was 12.4971 ppm centered at 4.691 ppm. Number of points acquired in the direct dimension was 2048 and 512 in the remote dimension. The relaxation delay was set to 2 s and the mixing time to 200 ms. Total correlation spectroscopy (TOCSY) using excitation sculpting for water suppression and MLEV17 for the TOCSY transfer. The spectral width in both dimensions was 12.4971 ppm centered at 4.691 ppm. Number of points acquired in the direct dimension was 1024 and 512 in the remote dimension. The relaxation delay was set to 1.5 s and the TOCSY mixing time to 90 ms. Correlation spectroscopy (COSY) using excitation sculpting for water suppression. The spectral width in both dimensions was 12.4971 ppm centered at 4.691 ppm. Number of points acquired in the direct dimension was 1024 and 512 in the remote dimension. Heteronuclear single quantum coherence (HSQC) with multiplicity editing. The spectral width for the proton dimensions was 11.001 ppm centered at 4.691 ppm and for the carbon dimension 169.9983 ppm centered at 80 ppm. Number of points acquired in the direct dimension was 1024 and 512 in the remote dimension. 2D HSQC-TOCSY using DISPI2 sequence for the TOCSY transfer. The spectral width for the proton dimensions was 11.001 ppm centered at 4.691 ppm and for the carbon dimension 169.9983 ppm centered at 80 ppm. Number of points acquired in the direct dimension was 1024 and 512 in the remote dimension. The relaxation delay was set to 1.5 s and the TOCSY mixing time to 80 ms.

### SPR Materials and Methods

SPR experiments were performed using the Nicoya Benchtop OpenSPR (Kitchener, ON, Canada) system with both high-capacity carboxyl sensors (SEN-AU-100-10-HC-COOH, lot no. SHE1101) and biotin sensors from the biotin–streptavidin sensor kit purchased from Nicoya (SEN-AU-100-10-STRP-KIT, lot no. SBE0505). All SPR experiments were performed with HBSS running buffer and without bovine serum albumin (BSA). Samples were diluted directly into the running buffer to minimize buffer clash and the background signal. Isopropyl alcohol (IPA; 80%) was used to remove bubbles from the system. Ligand immobilization for high-capacity carboxyl sensors was achieved using Nicoya’s Amine Coupling Kit (AMINE-10, lot no. KAD0816) that included 1-ethyl-3-carbodiimide (EDC) and *N*-hydroxysuccinimide (NHS) aliquots, 10 mM glycine-HCl, pH 2.0 (Nicoya, Reg-2.0, lot no. BAE1104), 10 mM sodium acetate, pH 5.0 (Nicoya, COOH-OPT-RK-10, lot no. KOE0826), and 1 M ethanolamine, pH 8.5 blocking solution (Nicoya, COOH-OPT-RK-10, lot no. RFE0826). Ligand immobilization for biotin sensors was achieved using 10 mM glycine-HCl, pH 2.0 (Nicoya, Reg-2.0, lot no. BAE1104) and recombinant His-tagged streptavidin (Amid Biosciences, cat. no. ST-301, lot no. 2109). Recombinant His-tagged human GFRAL (ACRO Biosystems, GFA-H52H3) expressed in HEK293 cells that contained a truncated Ser19–Glu351 domain was used for all SPR experiments and was confirmed via Western blot (SI, Figure S10). Initial validation was performed using commercially available full length recombinant GDF15 expressed in HEK293 cells (Abcam, ab302451).

HBSS pH 7.4 running buffer that was prepared in-house, and sonication was used to prime the SPR fluidics with a blank sensor chip installed for approximately 20 min. A high-capacity carboxyl sensor was then installed after the sensor was washed with deionized (DI) water and dried with compressed air. Buffer was allowed to flow over the carboxyl sensor for 2 min. This was followed by 80% IPA in repeated 150 μL injections until all bubbles over the sensor were removed. GFRAL was then immobilized onto the sensor surface by using the carboxyl sensor wizard. First, the sensor surface was conditioned with several 150 μL injections of 10 mM glycine HCl, pH 1.5, at a flow rate of 150 μL/min flow until a stable baseline was established. Next, aliquots of EDC and NHS were reconstituted separately each in 1 mL of Millipore water. These aliquots were then combined in a 1:1 volume:volume ratio and used for two 150 μL injections at 20 μL/min. Once this step was completed, lyophilized GFRAL protein was reconstituted in 625 μL of 10 mM sodium acetate, pH 5.0 buffer, to achieve a final concentration of 10 μg/mL. This was followed by four 150 μL injections of 10 μg/mL GFRAL each at a flow rate of 10 μL/min. Using this method, we ultimately achieved a total immobilization signal of 4000–6000 resonance units (RU) on channel 2 only. Finally, two 150 μL injections of 1 M ethanolamine, pH 8.5, were introduced at a flow rate of 20 μL/min to block the remaining reactive sites in the sensor. After GFRAL immobilization was complete, the system was reconditioned with glycine HCl, pH 1.5, and permitted to equilibrate for 20–30 min. Lyophilized GRASP peptides were then reconstituted at several concentrations in HBSS running buffer to minimize the background signal and buffer clashes. All concentrations of GRASP peptide were introduced in 150 μL injections at a rate of 20 μL/min. Glycine HCl, pH 1.5, was used to recondition after each injection.

Experiments performed with biotin sensors used the same running buffer and priming conditions as those used for the carboxyl sensors. A biotin sensor chip was installed in the system after it was washed with DI water and dried in compressed air. Buffer was allowed to flow over the biotin sensor for 2 min followed by bubble removal with 80% IPA; the IPA was added in repeating 150 μL injections until all bubbles have been removed from over the sensor. The sensor surface was then cleaned and conditioned using several 150 μL injections of 10 mM glycine HCl, pH 2.0, at a flow rate of 150 μL/min until a stable baseline was established. Streptavidin was diluted to 0.5 μM in HBSS running buffer and introduced to the biotin sensor via two 150 μL injections at a flow rate of 20 μL/min to achieve a maximum signal of 3000–4000 RU. The biotin-GRASP solution was then diluted to 50 μg/mL in HBSS running buffer and was applied to channel 2 of the streptavidin-coated sensor in two 150 μL injections at a flow rate of 10 μL/min to achieve a maximum signal of 500 RU. No additional blocking using a different biotin-tagged ligand was performed on channel 1. The system was allowed to equilibrate with the HBSS running buffer for 20–30 min at a flow rate of 20 μL/min. Lyophilized aliquots of GFRAL were reconstituted to several different concentrations in HBSS running buffer to minimize the background signal and buffer clashes. All concentrations of GFRAL were introduced via 150 μL injections at flow rates of 20 μL/min. Glycine HCl, pH 2.0, used to recondition the sensor after each injection. Additionally, following reconditioning, the sensor was recharged with streptavidin on both channels and with biotin–GRASP on channel 2 to re-establish full and consistent sensor surface coverage across the runs.

To obtain representative sensorgrams for use in calculating peptide binding kinetics, each system was permitted to reach binding equilibrium during injection at a given dose. This was required to calculate affinity constants based on the relationship between the equilibrium response and the peptide concentrations in a steady-state affinity model. Peptide dose–response evaluations were performed starting with the lowest concentration and moving forward to the highest concentration. Thus, if complete dissociation was observed, then regeneration buffer was not injected after each subsequent dose for a respective peptide. Otherwise, the system was regenerated with multiple injections of regeneration buffer before evaluation of the binding of the next peptide concentration. After the analysis of a given system was complete, a final GDF15 injection was performed to determine if any sensor (signal or RU) degradation occurred at any of the carboxyl sensors.

SPR binding curves generated from these data were evaluated kinetically by using TraceDrawer analysis software. High-resolution binding curves were loaded into the software and cropped appropriately with no added smoothing or data reduction performed to preserve the data integrity before further analysis. All curves were examined by using the same general evaluation specifications. First, the initial ligand interactions with the sensor and the subsequent completion of the ligand interaction were manually defined on the curves at the beginning of the association and dissociation periods, respectively. These two apparent changes in ligand concentration provided key interaction data points for the software that facilitated an analysis using a general one-to-one ligand–analyte fit model. The *K*_a_ and *K*_d_ values for all curves evaluated were analyzed using a global fit whereas the *B*_max_, of each curve was evaluated with a local fit; the BI was set to 0 because no bulk effect was observed. TraceDrawer software automatically evaluated all data and generated binding curves, together with relevant *X*^2^ and *U*-values for statistical error calculations.

### Flow Cytometric Evaluation of GRASPCy5 Interactions with GFRAL-Expressing HEK293 Cells

Flow cytometry measurements of GRASPCy5 binding were carried out on a BD Accuri C6 flow cytometer (BD Biosciences, Haryana, India). Panoply HEK293 cells stably overexpressing human GFRAL purchased from Creative Biogene (Shirley, NY, USA, CSC-SC006226) were used to evaluate GRASPCy5 surface binding compared to HEK293 wild-type cells. Cells were subcultured in T25 culture flasks (VWR, 10861-642) and incubated at 37 °C in a 5% CO_2_ incubator (ICO150, Memmert, Schwabach, Germany) to generate a monolayer at ∼95% confluency 48 h after subculturing. Cells were grown in Dulbecco’s Modified Eagle medium (DMEM, 1× with 4.5 g/L glucose, l-glutamine, and sodium pyruvate, Corning, 10-013CV, lot no. 11721014) supplemented with 10% fetal bovine serum (FBS, Avantor, 89510-186, lot no. 190B20). Medium was removed and replaced with various concentrations of fluorescent analyte in HBSS buffer at pH 7.4 and incubated at 37 °C in 5% CO_2_ for 30 min. The fluorescent analyte solutions were then removed by vacuum suction, and the cells were washed once with HBSS buffer. Cells were removed from the flasks with trypsin–EDTA solution (Sigma-Aldrich, SLCM3346), collected in HBSS buffer, and centrifuged at 600 rpm for 5 min. Supernatants were discarded, and cell pellets were washed again by resuspension in 5 mL of HBSS followed by centrifugation. The final washed pellet was resuspended in 1 mL of HBSS and filtered using a cell-strainer cap on a 5 mL polystyrene round-bottomed tube (Falcon, 352235) for flow cytometric analysis.

### Animal Experimental models

Adult male Sprague–Dawley rats (Charles River) weighing ∼250–270 g on arrival (*N* = 158) were housed under a 12 h:12 h light/dark cycle in a temperature- and humidity-controlled vivarium (23 ± 1 °C). Animals were fed *ad libitum* with a standard chow diet (Purina LabDiet 5001) with *ad libitum* access to tap water and kaolin pellets (Research Diets, K50001) for at least 5 days before the start of the experiment. All animals were naïve to experimental drugs and test treatments before the beginning of the experiment. Rats were habituated in single-hanging wire cages and received saline IP injections every day for 1 week before the onset of the experiment. All behavioral experiments involving GDF15 used a within-subject Latin Square design. Experiments with cisplatin were conducted using a pseudo-within-subject design. Each treatment round was separated for at least 72 h.

### Drugs and Route of Administration

For central administration directly into the CNS, GRASP, and GDF15 were infused in 1 μL volumes into the fourth ventricle (4th ICV). All systemic treatments were delivered by intraperitoneal (IP) injection. For central administration, GDF15 (human recombinant, Biovision, cat. 4569) was dissolved at a concentration of 30 pmol/μL in 100% dimethyl sulfoxide (DMSO). For systemic treatments, GDF15 was dissolved in a 5 mM acetate salt, 240 mM propylene glycol, and 0.007% polysorbate 20, in a pH 4 saline solution and injected at a dose of 20 μg/kg (1 mL/kg). For central administration, GRASP was dissolved in artificial cerebrospinal fluid (aCSF; Harvard Apparatus) and injected at 300 and 3000 pmol concentrations. Systemically delivered GRASP was dissolved in 0.9% saline and injected in a volume of 1 mL/kg at 30 and 100 nmol/kg. Cisplatin (Cis, *cis*-diammineplatinum dichloride, Sigma-Aldrich) was dissolved in 0.9% saline and administered at a dose of 6 mg/kg. The selective 5-HT_3_R antagonist Ondansetron (Tocris) was dissolved in 0.9% saline and IP was injected at 2 mg/kg (1 mL/kg). GDF15, GRASP, and/or Ondansetron were administered IP at 1 mL/kg, and cisplatin was administered IP at 6 mL/kg. Fluorescently tagged GDF15 and GRASP were dissolved in aCSF (300 pmol/μL); 1 μL was infused into the lateral ventricle (LV ICV).

### Stereotactic Surgery

Drug instillation into the CNS was facilitated by a cannula placement. Rats were anesthetized by IP administration of ketamine (90 mg/kg, Butler Animal Health Supply), xylazine (2.7 mg/kg, Anased), and acepromazine (0.64 mg/kg, Butler Animal Health Supply). A 26-gauge guide cannula (8 mm, 81C3151/Spc, Plastics One) directed at the fourth ventricle (2.5 mm anterior to the occipital suture and 7.2 mm ventral to skull surface) and at the lateral ventricle (0.9 mm anterior to Bregma, 1.6 mm lateral to the midline and 3.5 mm ventral to skull surface) was implanted and affixed to the skull with screws and dental cement, as previously described. Metacam (meloxicam, 2 mg/kg, Midwest Veterinary Supply, IP) was administered daily subcutaneously immediately after surgery and for two consecutive days thereafter. All rats were given 1 week to recover from surgery. To confirm cannula placement, the 5-thio-d-glucose test was conducted, as previously described.^37^ All rats passed verification tests and were therefore included in the experiment.

### Assessment of GRASP/GFRAL Colocalization in the AP/NTS

Rats (*n* = 3, 300–330 g) received an intraventricular infusion of GRASP555. Two hours later, the animals were deeply anesthetized with ketamine/xylazine/acepromazine as described above and transcardially perfused with phosphate-buffered saline (PBS, 0.1 M, pH 7.4; Boston Bioproducts), followed by 4% paraformaldehyde (PFA) in PBS. Brains were removed, postfixed in 4% PFA for 48 h, and then stored in 30% sucrose for 2 days. Brains were subsequently frozen in cold hexane and stored at −20 °C for further processing. The 15 micrometer-thick frozen coronal sections containing the AP/NTS were cut in a cryomicrotome (CM3050S, Leica Microsystem), collected, and immediately mounted onto glass slides (Superfrost Plus, VWR). Slides were stored at −20 °C for further processing. Immunofluorescence staining of brain sections was conducted primarily as previously described.^15^ Briefly, after antigen retrieval (incubation in hot citrate solution [Vector, H-3000] for 1 h), slides were rinsed with 0.1 M PBS containing 0.1% triton X-100 (0.1% PBST) for 5 min and then incubated in 0.1% PBST containing 5% normal donkey serum (NDS) for 30 min. This was followed by overnight incubation with the sheep anti-GFRAL antibody (1:500, Thermo Fisher, PA5–47769, in 0.1% PBST). After washing with 0.1% PBST three times for 15 min each, sections were incubated with the secondary donkey antisheep Alexa Fluor 488 for 2 h at room temperature. After final washing (as above), the sections were coverslipped with antifade mounting medium and DAPI (Vector Laboratories). GRASP555-, GFRAL-, and DAPI-positive cells were visualized by fluorescence microscopy (20×; Keyence BZ-X800).

### Effects of GRASP on GDF15-Induced Anorexia and Pica

To evaluate the impact of GRASP on GDF15-induced anorexia and nausea (proxied by kaolin ingestion) in rats (*n* = 10, 400–450 g), GRASP (300 and 3000 pmol), or vehicle control was administered centrally immediately before intraventricular infusion of GDF15 (30 pmol). A second cohort of rats (*n* = 12, 500–550 g) received intraventricular doses of GRASP (30 and 100 nmol/kg) or vehicle immediately before systemic administration of GDF15 (20 μg/kg). In both experiments, food intake and kaolin consumption were measured at 3, 6, and 24 h postinjection.

### Effects of GRASP on Cisplatin-Induced Nausea and Anorexia

Using a similar paradigm, the impact of GRASP on cisplatin-induced nausea and anorexia was assessed. First, rats (*n* = 35, 330–380 g) received a single injection of GRASP (100 nmol/kg) or vehicle followed 15 min later by a second injection containing cisplatin or saline control. Food intake and kaolin consumption were measured at 3, 6, and 24 h postinjection.

### Impact of Coadministration of GRASP and Ondansetron on Cisplatin-Induced Malaise

Rats (*n* = 98, 300–330 g) received IP injections of GRASP (100 nmol/kg), Ondansetron (2 mg/kg), both GRASP and Ondansetron together, or saline control 15 before systemic administration of cisplatin. Six hours later, rats received a second injection of GRASP or vehicle control. Food and kaolin intake were measured at 3, 6, and 24 h postinjection.

### Statistical Analysis

Food intake and kaolin consumption were analyzed using repeated measures 2 × 3 ANOVAs, 2 × 2 ANOVAs, or one-way ANOVAs followed by Tukey’s *post hoc* tests. All data were expressed as means ± SEM. For all statistical tests, a *p*-value less than 0.05 was considered significant. All data were analyzed using Prism 9 GraphPad Software (San Diego, California).

### Ethics Statement

All animal procedures were conducted as approved by the Institutional Care and Use Committee of the University of Pennsylvania and performed according to the guidelines determined by the National Institutes of Health.

## Supporting Information Available

Experimental methods and additional data on peptide synthesis, purification, characterization, *in vitro* assays, and *in vivo* experiments (PDF)
GRASP NMR data (PDB)
Molecular docking data (ZIP)
Data for GRASP NMR chemical shift data (TXT)
Data for GRASP NMR constraints (TXT)

## Supplementary Material

jm3c00667_si_001.pdf

jm3c00667_si_002.pdb

## Abbreviations

aCSF: artificial cerebrospinal fluid
AP: area postrema
BID: twice a day
BMRB: Biological Magnetic Resonance Databank
BSA: bovine serum albumin
CD: circular dichroism spectroscopy
CINV: chemotherapy-induced nausea and vomiting
CNS: central nervous system
CSF: cerebrospinal fluid
Cy5: sulfo-cyanine5
DBCO: dibenzocyclooctyne
DCM: dichloromethane
DMEM: Dulbecco’s Modified Eagle Medium
DMF: *N*,*N*-dimethylformamide
ECD: extracellular domain
ESI-MS: electrospray ionization mass spectrometry
ESMS: electrospray mass spectrometry
FBS: fetal bovine serum
GDF15: growth differentiation factor 15
GDNF: glial cell-derived neurotrophic factor
GFRAL: GDNF family receptor alpha-like
GRASP: GFRAL receptor antagonist
HBSS: Hank’s balanced salt solution
HEK: human embryonic kidney cells
HPLC: high-performance liquid chromatography
ICV: intracerebroventricular
IP: Intraperitoneal
*K*_a_: association constant
KAX: ketamine
*K*_D_: binding affinity
LV: lateral ventricle
MOE: Molecular Operating Environment
NDS: normal donkey serum
NMR: nuclear magnetic resonance
NTS: nucleus tractus solitarius
OND: Ondansetron
PBS: phosphate buffered saline
PDB: Protein Data Bank
PFA: paraformaldehyde
RET: rearranged during transfection
RFU: relative fluorescence units
RU: resonance units
SPAAC: strain promoted alkyne–azide cycloaddition
SPR: surface plasmon resonance
TFA: trifluoroacetic acid
TGF-β: transforming growth factor-beta
TIPS: triisopropylsilane
TBTA: tris(benzyltriazolylmethyl)amine
wt: wild-type.
