# Supplementary material for: Creation of a Peptide Antagonist of the GFRAL–RET Receptor Complex for the Treatment of GDF15-Induced Malaise
Source: J Med Chem. 2023 Jul 28;66(16):11237–49. doi: 10.1021/acs.jmedchem.3c00667 (PMC10461225; doi:10.1021/acs.jmedchem.3c00667)
Supplement: Supplementary file 1 — jm3c00667_si_001.pdf [file jm3c00667_si_001.pdf]

SUPPLEMENTARY INFORMATION

Creation of a Peptide Antagonist of the GFRAL-RET Receptor Complex for the Treatment of GDF15-Induced Malaise

Tito Borner,[2,3,‡] Ian C. Tinsley,[1,‡,^] Brandon T. Milliken,[1] Sarah A. Doebley,[2] Nicholas R. Najjar,[1]

Deborah J. Kerwood,[1] Bart C. De Jonghe,[2,3,*] Matthew R. Hayes,[2,3,*] and Robert P. Doyle,[1,4,*]

[1]Syracuse University, Department of Chemistry, 111 College Place, Syracuse, NY 13244 (USA)

[2]University of Pennsylvania, Department of Biobehavioral Health Sciences, School of Nursing, Philadelphia, PA 19104 (USA)

[3]University of Pennsylvania, Department of Psychiatry, Perelman School of Medicine, Philadelphia, PA 19104 (USA)

[4]State University of New York, Upstate Medical University, Departments of Medicine and Pharmacology, Syracuse, NY 13245 (USA)

[^] Current address: Department of Chemistry, Indiana University, Bloomington, IN 47405 (USA)

Corresponding author: rpdoyle@syr.edu

Table of Contents **Page**

Table of Contents	Page
Figure S1-2.	#S3
Figure S3-4.	#S4
Figure S5-6.	#S5
Figure S7-8.	#S6
Figure S9.	#S7
Figure S10.	#S8
Table S1.	#S9
Figure S11.	#S8
Figure S12.	#S10
Figure S13.	#S11

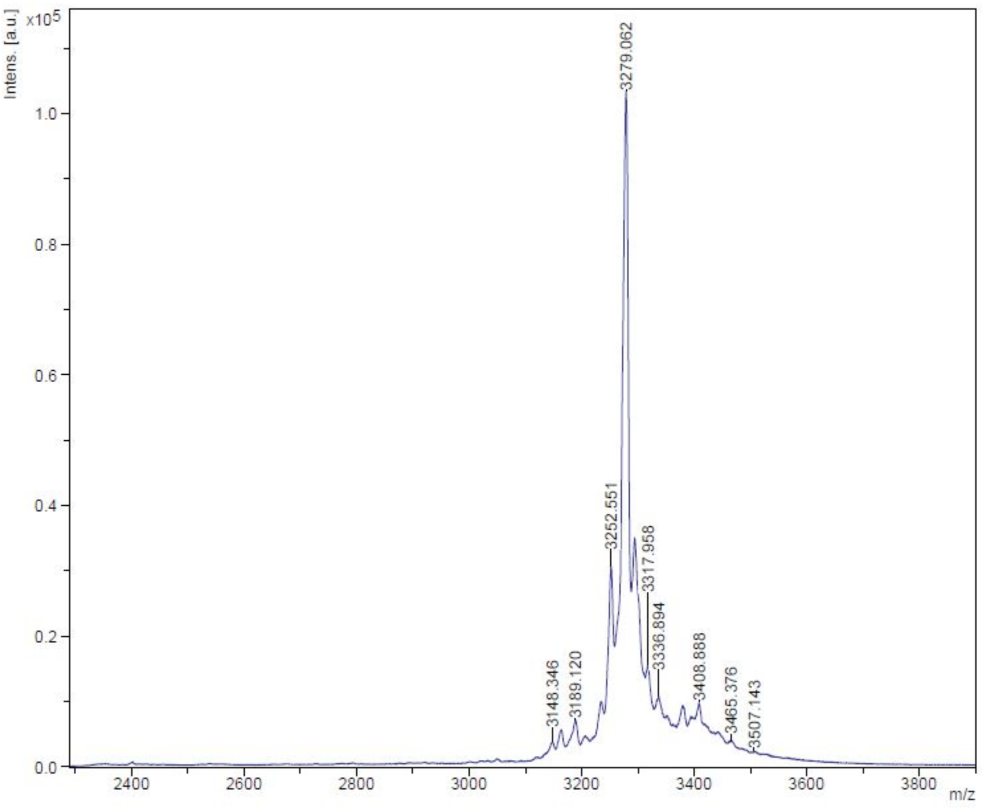

Figure S1. GRASPK2azido mass identification via MALDI-Tos MS.

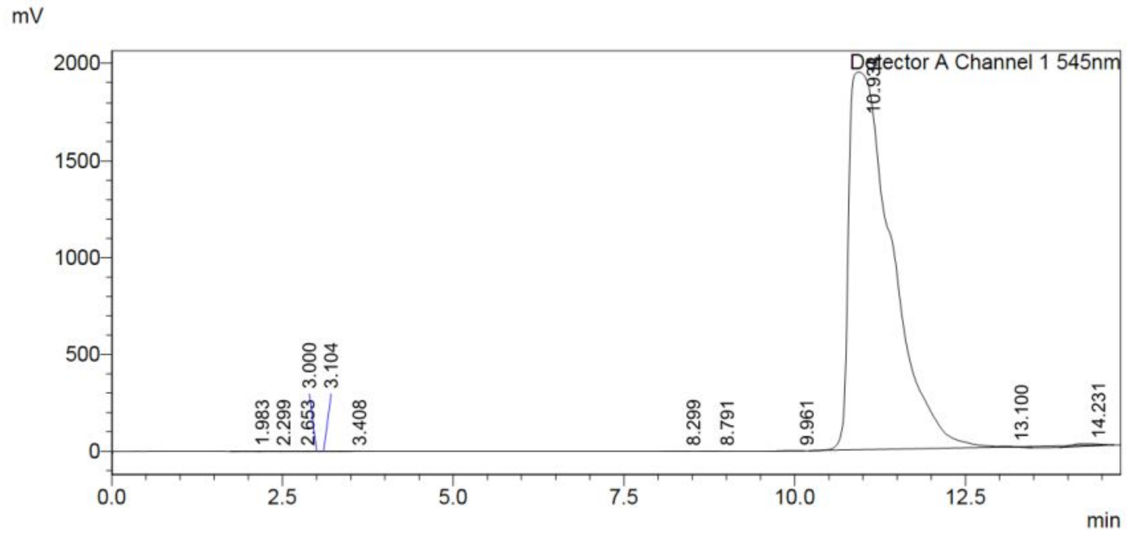

Figure S2. GRASP555 Purification: RP-HPLC Trace (Shimadzu Prominence HPLC using a C18 column (Eclipse XDB-C18 5µm, 4.6 x 150 mm) (RP-HPLC, from 1% CH_3OH/H_2O + 0.1% TFA to 90% CH_3OH/H_2O + 0.1% TFA in 25 min)) showing GRASP-555 product at 10.9 min. Purity 99%.

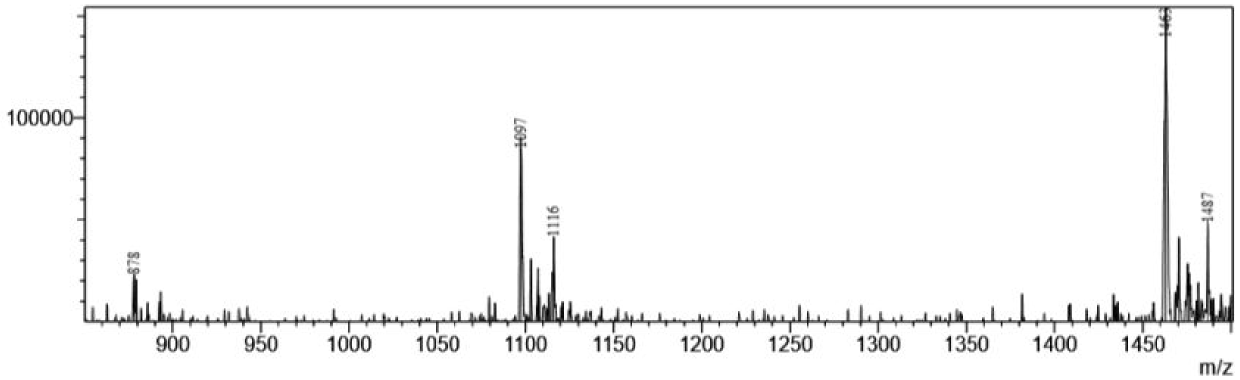

Figure S3. GRASP555 mass identification: ESI-MS (Shimadzu LCMS-8040) of GRASP-555, expected m/z = 4386, observed m/z = $[M+2H^{+}+Na^{+}]^{+3}$: 1487, $[M+3H]^{+3}$: 1463, $[M+4H^{+}+H_2O]^{+4}$: 1116, $[M+4H]^{+4}$: 1097, $[M+5H]^{+5}$: 878.

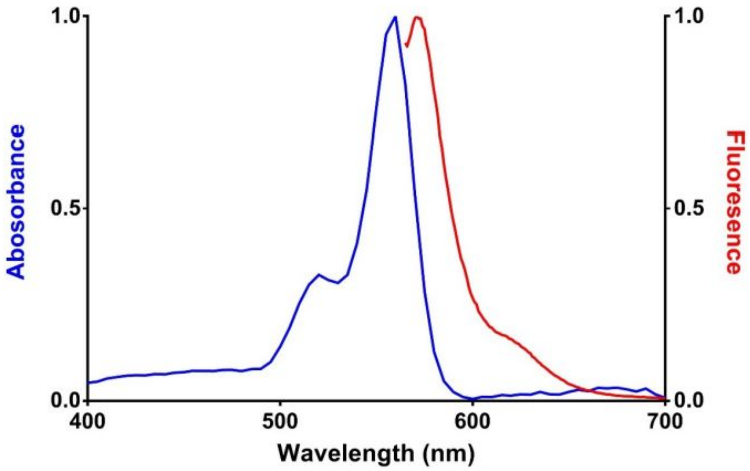

Figure S4. GRASP-555 electronic absorption spectra were obtained on a Varian Cary 50 Bio spectrophotometer in a 2mL quartz cuvette between 400 nm – 700 nm in aqueous acetonitrile.

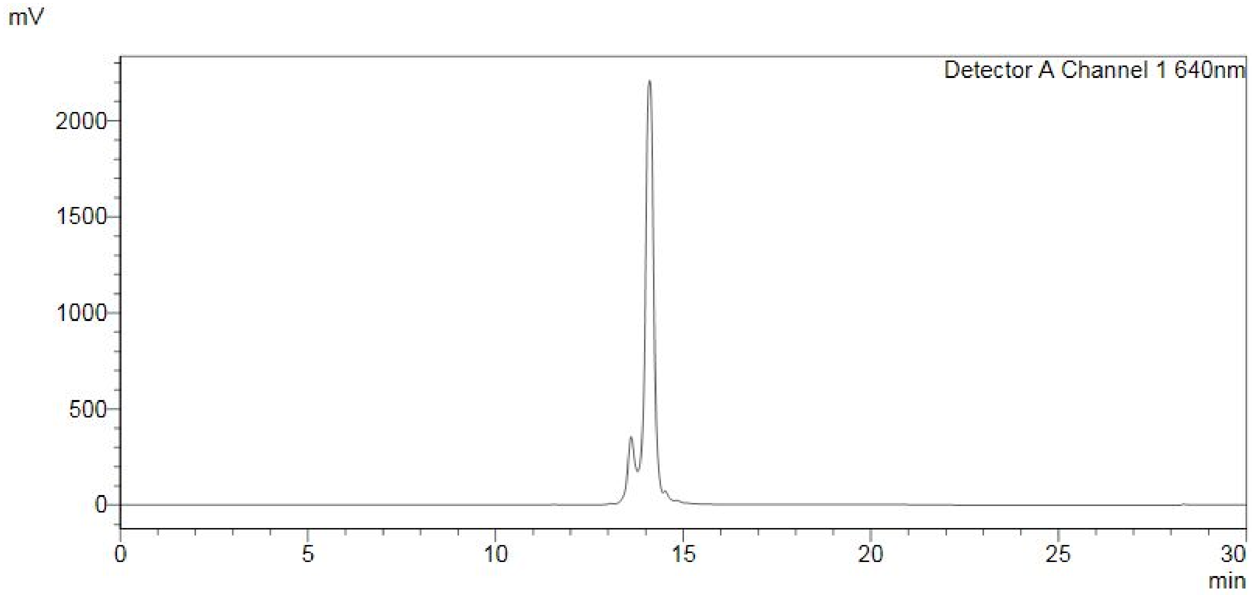

Figure S5. GRASPCy5 purification: RP-HPLC Trace (Shimadzu Prominence HPLC using a C18 column (Eclipse XDB-C18 5μm, 4.6 x 150 mm) (RP-HPLC, from 1% ACN/H2O + 0.1% TFA to 90% ACN/H2O + 0.1% TFA in 25 min)) showing GRASPCy5 product at 14 min. Purity 95%

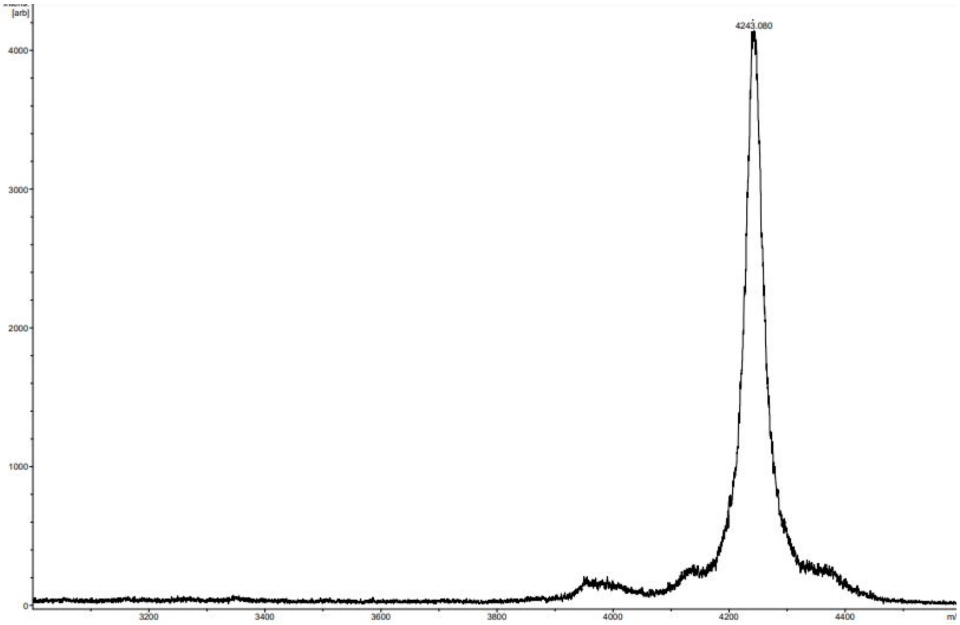

Figure S6. GRASPCy5 mass identification via MALDI-Tof MS.

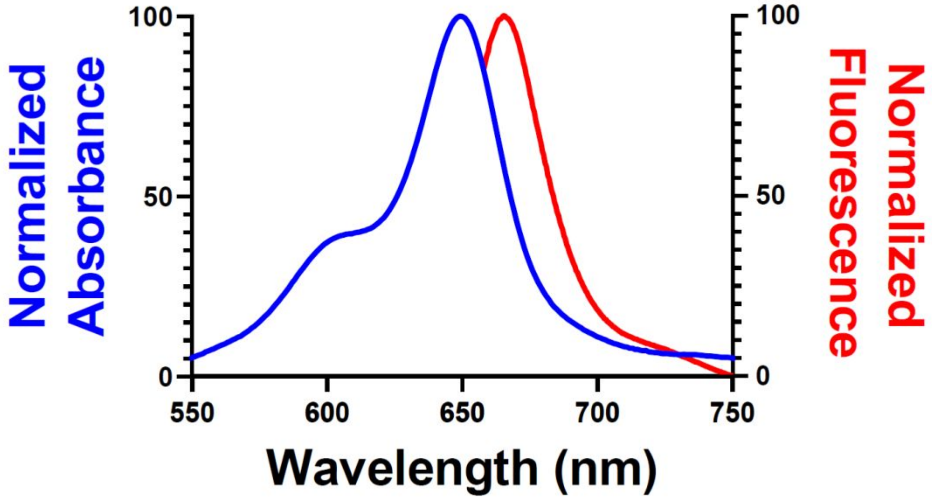

Figure S7. Absorption and Fluorescent spectroscopy of GRASPCy5: Electronic absorption spectra were obtained using an Agilent Technologies Cary Series UV-Vis Spectrophotometer scanning between 550 nm – 750 nm in water, λmax = 649 nm. Fluorescence spectra was obtained using an Agilent Cary Eclipse Fluorescence Spectrophotometer scanning between 650 nm – 750 nm in water.   Excitation at 649 nm; Emission maxima at 665.5 nm.

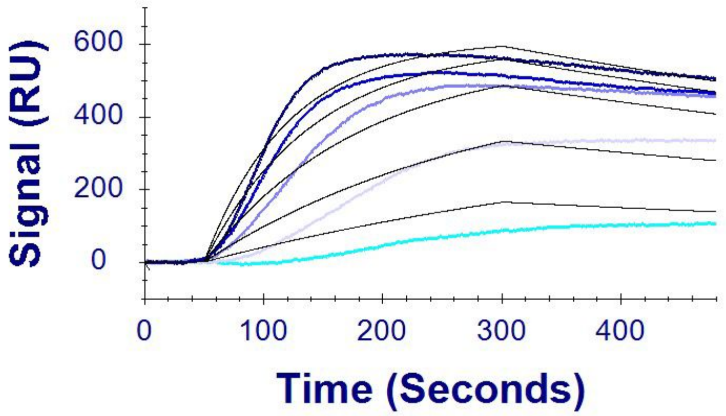

Figure S8. Commercial recombinant GDF15 SPR sensorgram: Overlain black lines represent TraceDrawer kinetic evaluation yielding a K_D = 6.02 nM.

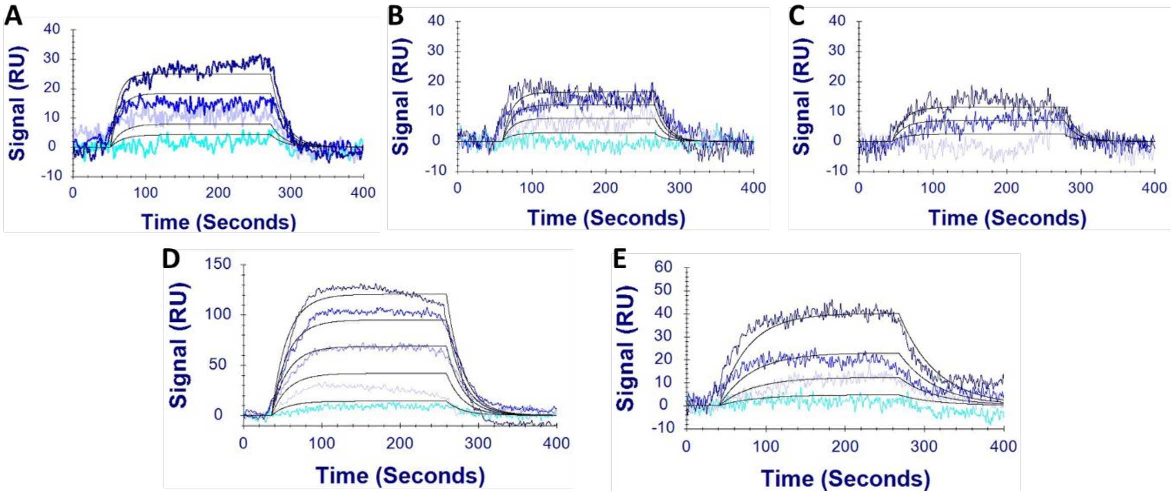

Figure S9. Surface Plasmon Resonance (SPR) carboxyl sensor GSP dose response Sensorgrams with overlain TraceDrawer kinetic evaluation curves of best fit. Relevant K_D values recorded in sequence table. (A) GSP01 (GRASP), (B) GSP02, (C) GSP03, (D) GSP04, (E) GSP05. See Table S1 for sequences.

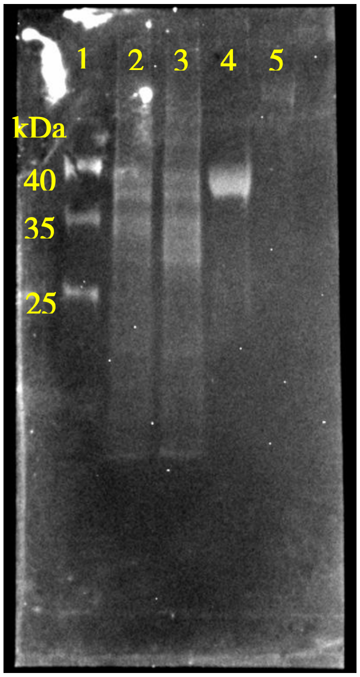

Figure S10. GFRAL western Blot. Lane 1: Spectra™ Multicolor Broad Range Protein Ladder (Catalog number: 26634). Lane 2: Lysed WT HEK293 cells. Lane 3: Lysed GFRAL overexpressing HEK293 cells. Lane 4: GFRAL Control. Lane 5: RET Control. Western Blot ran using 1X electrophoresis buffer on Invitrogen NuPAGE 12% Bis-Tris Gel (Cat#: NP0342BOX). Transfer to an Invitrogen iBlot 2 NC mini stack nitrocellulose membrane. Primary antibody: Invitrogen GFRAL Polyclonal antibody Rb IgG (Cat#: PA5-24545). Secondary antibody: Abcam Goat pAb to Rb IgG (HRP) (Cat#: ab205718). Imaged using ThermoScientific SuperSignal West Pico Plus Chemiluminescent substrate.

Peptide	Sequence	AA	MW (Da)	K_D (μM)
GRASP	TKEELIHAHADPMVLIQKTDTGVSLQTYD	29	3280	179
GSP02	EDDVSFQKLDDNVRYHTLRK	20	2504	2350
GSP03	DDDLSFQKLDDNVYYHLLRK	20	2522	64800
GSP04	KLDDNVYYHLLRK	13	1702	9010
GSP05	KPMVLIQKTDTGVSLQTYD	19	2162	1380

Table S1. Polypeptide GFRAL antagonists. Peptide sequences were derived from the amino acid sequence of the native GFRAL ligand, GDF15 with a focus on critical binding regions that were conserved across other GDNF receptors and their ligands. K_D values calculated from carboxyl sensor chip SPR experiments using TraceDrawer software 1:1 global kinetic fit. (Abv. AA = amino acid; MW = molecular weight). K residue at position 2 of GRASP is ε-azido lysine).

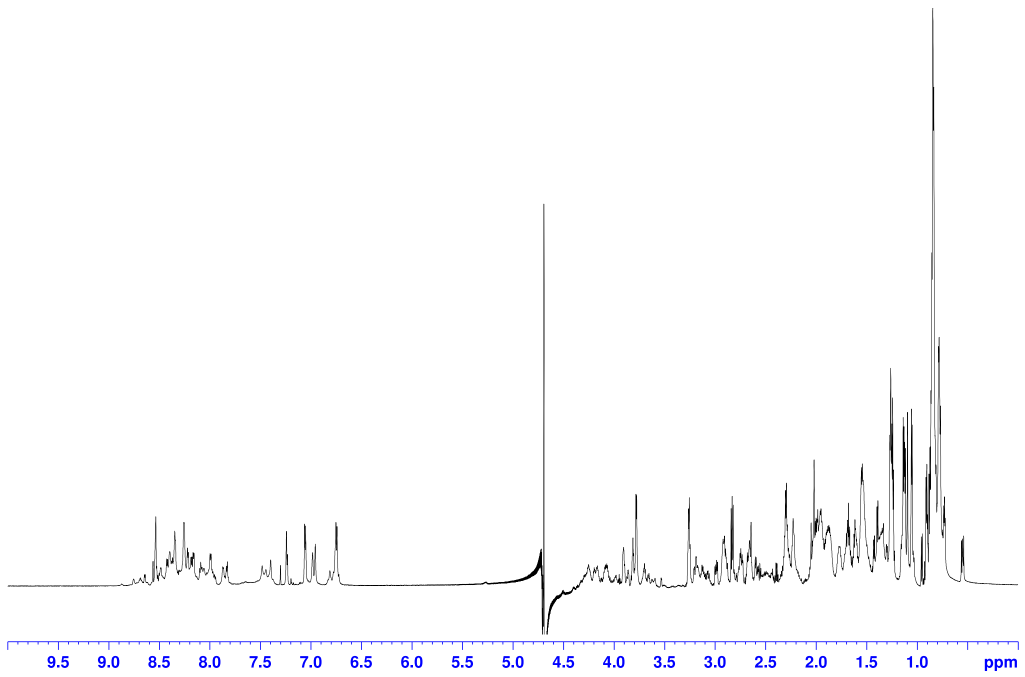

Figure S11. 1D proton NMR (800 MHz; cryoprobe) of GRASP (300 μM) with water suppression using watergate pulse program performed at 25 °C. Structure deposited in the BMRB as Entry ID 51672.

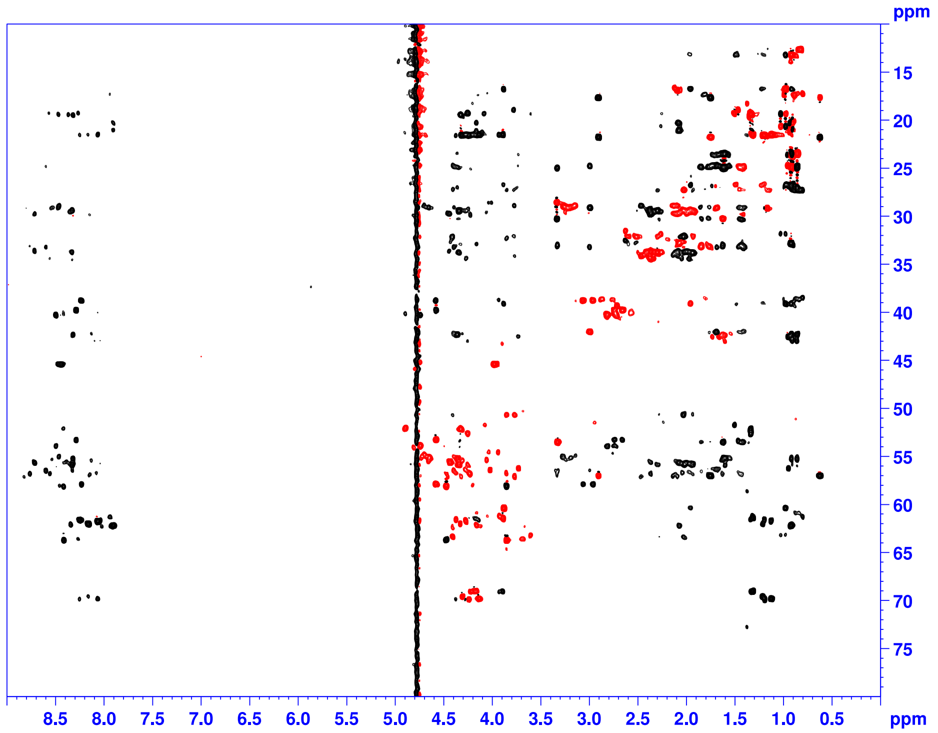

Figure S12. 2D NMR (800 MHz; cryoprobe) edited 1H-13C HSQC-TOCSY of GRASP (300 μM) performed at 25 °C. Structure deposited in the BMRB as Entry ID 51672.

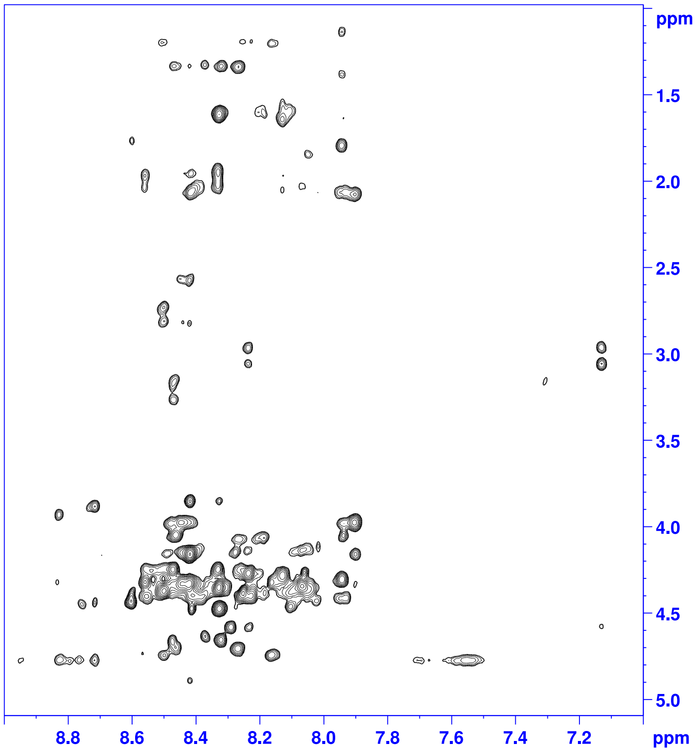

Figure S13. NOESY NMR (800 MHz; cryoprobe): NH region of the NOESY spectrum of GRASP (300 μM) performed at 25 ^{o}C. Structure deposited in the BMRB as Entry ID 51672.
